# Role of miR-506 in ulcerative colitis associated with primary sclerosing cholangitis

**DOI:** 10.1038/s41598-021-89631-4

**Published:** 2021-05-12

**Authors:** Agnieszka Kempinska-Podhorodecka, Monika Adamowicz, Ewa Ostrycharz, Mateusz Chmielarz, Maciej Wójcicki, Piotr Milkiewicz, Malgorzata Milkiewicz

**Affiliations:** 1 Department of Medical Biology, Pomeranian Medical University, 70-111 Szczecin, Poland; 2 Liver and Internal Medicine Unit, Medical University of Warsaw, 02-097 Warsaw, Poland; 3 European Reference Network (ERN) Rare-Liver, Warsaw, Poland; 4 Translational Medicine Group, Pomeranian Medical University, 70-111 Szczecin, Poland

**Keywords:** Molecular biology, Gastroenterology

## Abstract

Primary sclerosing cholangitis (PSC) is commonly accompanied by ulcerative colitis (UC). MicroRNA-506 modulates expression of genes which are essential for sphingosine-mediated signaling pathway and intestinal mucosa protection. We investigated whether miR-506 and its target genes are involved in phenotypic presentations of colonic inflammation and/or neoplasia. We analyzed serum and colon tissue samples collected from patients with PSC, PSC with concurrent UC (PSC + UC), UC alone, and healthy controls (n = 10 each). MiR-506 was substantially upregulated in ascending colons of PSC and PSC + UC patients, in contrast to sigmoid colons of PSC and UC patients. Upregulation of miR-506 was associated with inhibition of SPHK1, AE2, InsP3R3, and p53. Colonic suppression of miR-506 presented in UC was accompanied by substantially increased DNMT1, SPHK1, and S1P lyase expressions. A functional in vitro analysis in Caco-2 cells showed that the induction of miR-506 activity by miR-506 mimic or GDCDA bile acid suppressed, whereas inhibition of miR-506 by miR-506 inhibitor or lipopolysaccharide (LPS) upregulated the expression of the examined target genes. A different phenotypic presentation of colitis may be related to miR-506 expression. In ascending colons with PSC + UC, upregulation of miR-506 may result in failure of bicarbonate secretion and inhibition of p53, which predisposes to pro-tumorigenic transformation. In contrast, downregulation of miR-506 enhances S1P production, leading to pro-inflammatory signaling.

## Introduction

Primary sclerosing cholangitis (PSC) is a chronic cholestatic liver condition affecting both small and large bile ducts, leading to inflammation, fibrosis, and—in a proportion of patients—liver cirrhosis. The strong association between PSC and IBD, and ulcerative colitis (UC) in particular, is well established. In contrast to UC without PSC, a majority of patients with both conditions more frequently experience inflammation in the right (i.e., ascending) colon, with a significant proportion of patients exhibiting backwash ileitis and rectal sparing^1^. Moreover, patients with concomitant PSC have an increased risk of developing primary bile duct cancer and colorectal cancer (CRC)^2^. Recently, the mechanism of CRC tumorigenesis has been linked to microRNAs (miRNAs), a class of small non-coding RNAs that modulate gene expression. MiRNAs may possess either tumor-suppressive or oncogenic activity, depending on their target genes^3^. Similarly, diseases involving excessive or uncontrolled inflammation are accompanied by dysregulation of miRNAs^4^.

MiR-506 is a miRNA that was recently shown to directly target genes involved in various biological processes, including tumorigenesis, cell proliferation, metastasis, suppression of epithelial-to-mesenchymal transition, and immune response^5^. MiR-506 has been found to play a number of pivotal roles in several cancer types, as it acts either as a tumor suppressor in ovarian or lung cancer^6^ or as an oncogene in melanomas^7^ Although the downregulation of miR-506 was observed in human CRC and CRC cell lines^8^, little is known about the miR-506-dependent intrinsic regulatory mechanisms in colorectal cancer.

Sphingosine-1-phosphate (S1P), a bioactive sphingolipid, plays fundamental roles in the cancer microenvironment as it inhibits apoptosis, promotes oncogenesis, and augments inflammation^9^. S1P levels are regulated through the balance between its synthesis (initiated by sphingosine kinases (SPHKs) and its temporary deactivation by sphingosine-1-phosphate phosphatase (SPP) or permanent breaking catalyzed by S1P lyase (SPL)^10^. An association has been found between the overexpression of miR-506 and the inhibition of sphingosine kinase 1 (SPHK1)^11^. Expression of SPHK1 is increased in several types of human tumors, and enhancement of its expression correlates with disease progression and reduced patient survival^12^. Solid tumors are often oxygen insufficient, and hypoxia is known to induce SPHK1 expression, which promotes neovascularization of tumors^10^. Moreover, SPL has been reported to potentiate apoptosis via a p53-dependent pathway^13^, and the loss of S1P lyase activity promotes neoplastic transformation and tumorigenesis in different cancer types^14^.

Downregulation of miR-506 is associated with significant induction of DNA methyltransferase 1 (DNMT1) expression^8^ that enhances promoter methylation of the tumor suppressor gene phosphatase and tensin homolog (PTEN)^15^. In many tumor types, genetic alterations of PTEN enhance tumorigenesis, and may determine aggressive clinicopathological behavior of a tumor^16,17^. The overexpression of PTEN has been shown to induce p27^Kip1^ in intestinal cells^18^. The association between the miR-506-related DNMT1/PTEN axis and CRC incidences in PSC patients has not been examined.

Furthermore, miR-506 has been demonstrated to downregulate two important factors that play a key role in HCO3- secretion into intestinal lumen and bile: the Cl-/HCO3- exchanger anion exchange protein 2 (AE2) and inositol-1,4,5-trisphosphate-receptor (InsP3R3)^19^. HCO3- secretion is essential for duodenal mucosa protection, as colonic bicarbonate secretion is promoted by the release of Ca2 + via InsP3R3^20^. Bile acid toxicity in humans has been shown to be highest when AE2 expression is suppressed^21^.

We investigated a potential role of miR-506 in the context of pathological changes, such as inflammation or neoplasia, that frequently manifest in colons of PSC patients. Given that PSC patients are characterized by different phenotypic presentation of colitis and a higher predisposition to colon cancer as compared to patients with UC alone, we analyzed the expression of miR-506 and its target genes in the ascending and sigmoid colons of PSC patients.

## Results

**Figure 1:**
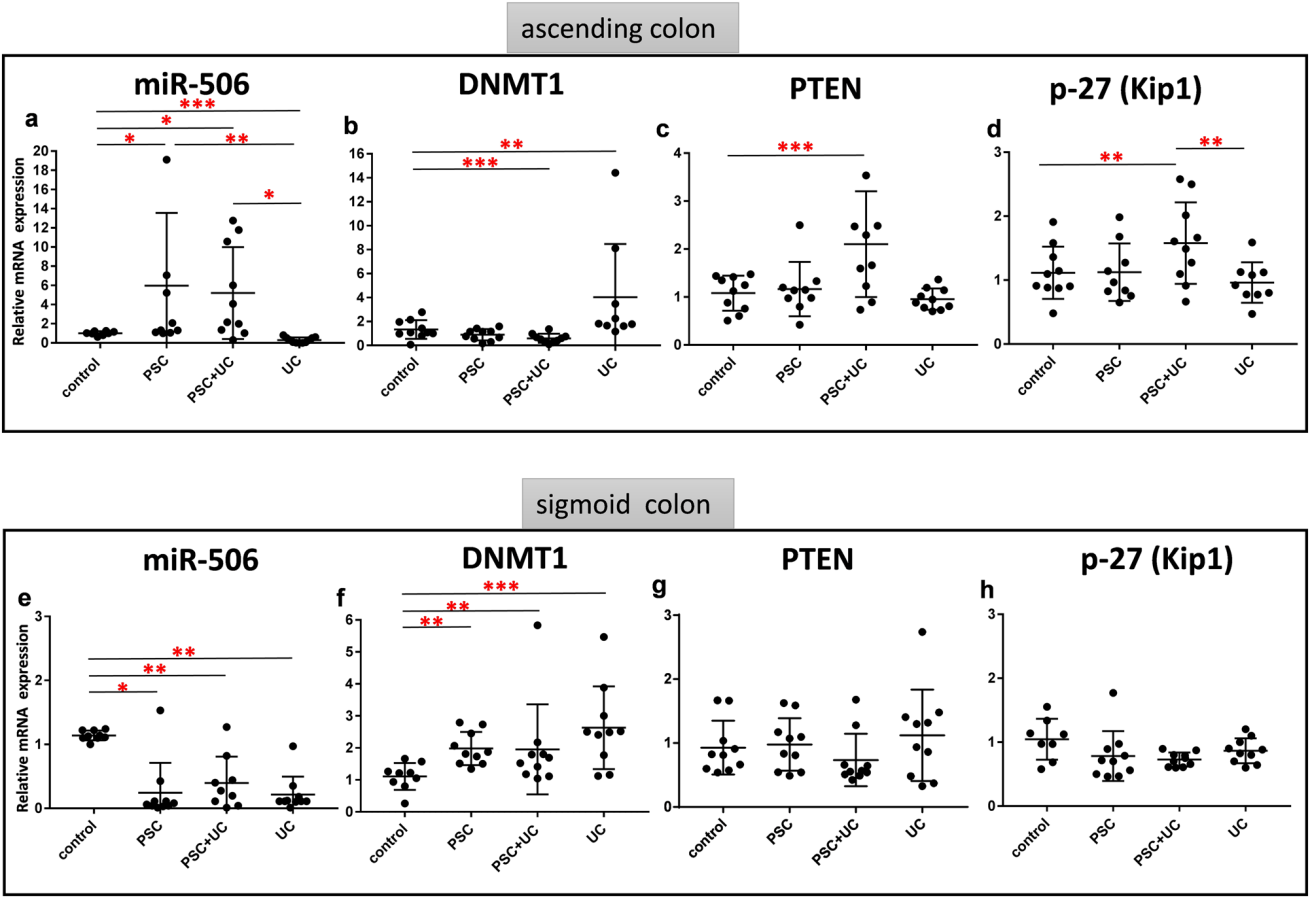
Expression of microRNA-506 (miR-506), DNA-methyltransferase-1 (DNMT1), phosphatase and tensin homolog (PTEN), and p27(Kip1) mRNAs in large intestines of the patients. Relative expression levels of miR-506 **(a,d)**, DNMT1 **(b,e)**, PTEN **(c,f)**, p27Kip1**(d,g)** in ascending **(a–d)** and sigmoid colons **(e–h)** of controls (n = 10), PSC (n = 10), PSC + UC (n = 10), and UC (n = 10). The expression of all genes are normalized to an endogenous reference (miR-191 for miR-506, or 18S rRNA for other genes) and presented as a relative fold change to controls according to the comparative Ct method (2^−ΔΔCt.^). Dots illustrate each patient and lines the mean plus interquartile range (IQR). Statistics: one-way ANOVA followed by Fisher’s PLSD test within the groups; *indicates p-value < 0.05, ** p-value p < 0.01, *** p-value < 0.001.

The expression of all genes are normalized to an endogenous reference and presented as a relative fold change to healthy controls. First, we analyzed the expression of miR-506 and its downstream targets, the DNMT1 and PTEN genes. In the ascending colon, miR-506 expression was substantially increased in both PSC patients (5.4-fold difference vs. controls, *p* = 0.02) and PSC + UC patients (4.7-fold difference vs. controls, *p* = 0.02, Fig. 1a). In PSC + UC patients, upregulation of miR-506 was accompanied by decreased levels of DNMT1 (58% reduction vs. controls, *p* = 0.0001, Fig. 1b, Spearman’s rank correlation Rho: -0.31, p = 0.05, Table 1), and increased levels of PTEN mRNA (1.9-fold difference vs. controls, *p* = 0.001 (Fig. 1c). In PSC patients, levels of DNMT1 and PTEN mRNA were not changed in comparison to controls (Fig. 1b,c). On the contrary, miR-506 expression was significantly suppressed when compared to controls in the sigmoid colons of PSC patients with or without UC (decreased by 78% in PSC and 60% in PSC + UC), and in both parts of the colons of UC patients (70% in ascending colons and 80% in sigmoid colons; Fig. 1a,e). This was accompanied by an increase in DNMT1 mRNA levels (1.4-fold in PSC, 1.3-fold in PSC + UC, 9.8-fold in UC ascending colons, and 3.5-fold in UC sigmoid colons, Fig. 1b,f). Correlations between miR-506 levels and SPHK1 (Rho − 0.48 *p* = 0.002) and DNMT1(Rho − 0.40 *p* = 0.01) in sigmoid colons are presented in Table 1. In terms of PTEN expression, there were no statistically significant differences between groups (Fig. 1g).

**Table 1:** Spearman’s rank correlations for miR-506, SPHK1, and DNMT1 mRNA expressions analyzed in colon tissue of PSC (n = 10), PSC + UC (n = 10), and UC (n = 10) patients.

Parameters		Rho	*p*-value*
**Ascending colon**			
miR-506	SPHK1	−0.308	**0.05**
	DNMT1	−0.246	0.1
SPHK1	DNMT1	0.841	**0.0001**
**Sigmoid colon**			
miR-506	SPHK1	−0.483	**0.002**
	DNMT1	−0.402	**0.01**
SPHK1	DNMT1	0.536	**0.0005**

*miR-506* microRNA-506, *SPHK1* sphingosine-kinase-1, *DNMT1* DNA-methyltransferase-1, *mRNA* messenger RNA.

*Value in boldface indicates statistically significant difference (Spearman’s rank correlation).

Given that PTEN activity leads to the induction of p27(Kip1) expression, which in turn can negatively regulate the transition through the cell cycle, we investigated the expression of p27(Kip1). We observed the upregulation of p27(Kip1) mRNA only in ascending colons of PSC patients with concurrent UC (1.6-fold vs. controls, *p* = 0.01, and 1.5-fold vs. PSC, *p* = 0.04; Fig. 1d,h).

**Figure 2:**
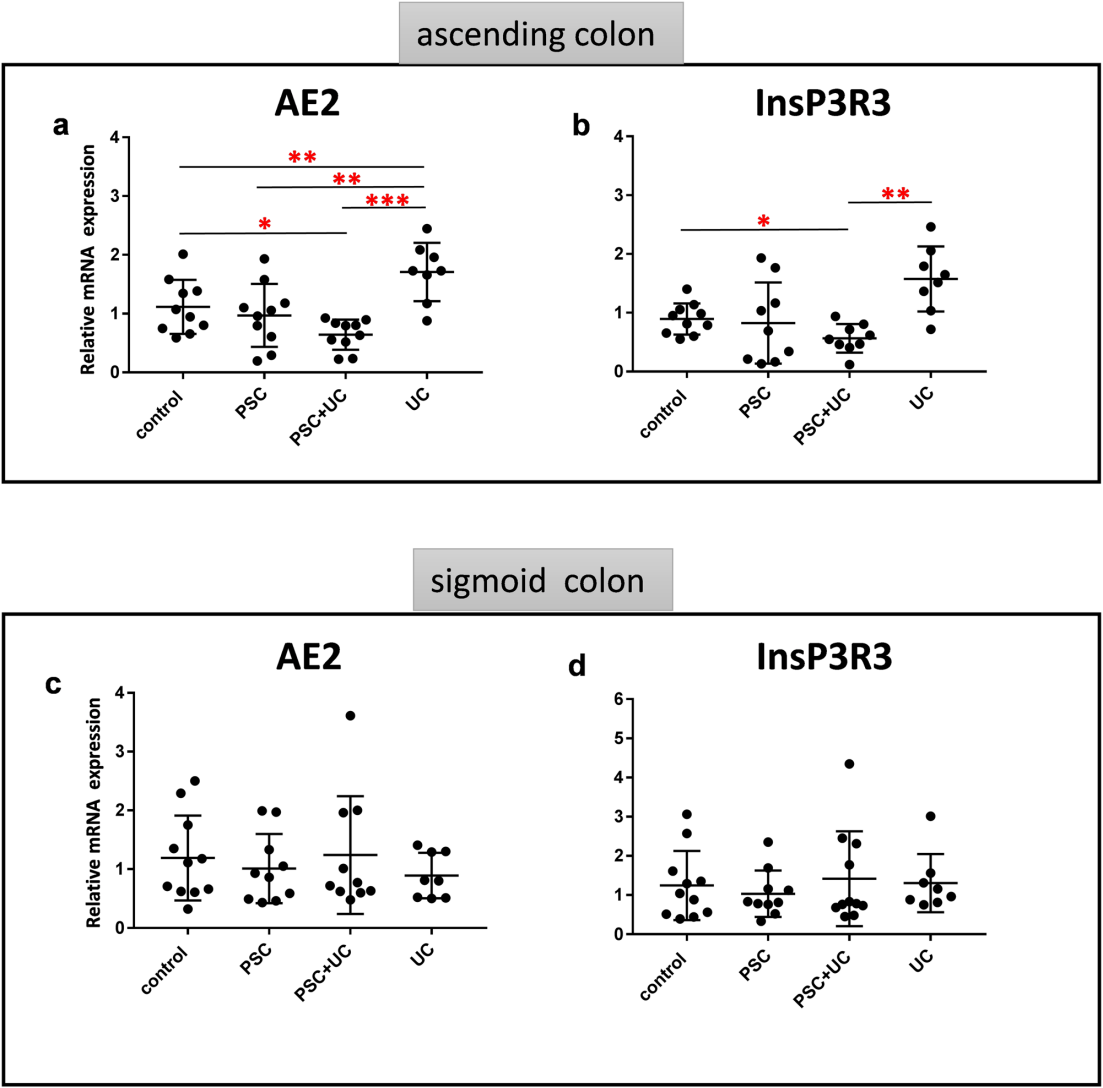
Expression of AE2 and InsP3R3 mRNA in large intestines of the patients. Relative expression levels of anion exchange protein 2 (AE2) **(a,c)** and inositol-1,4,5-trisphosphate-receptor (InsP3R3) **(b,d)** in ascending **(a,b)** and sigmoid colons **(c,d)** of controls (n = 10), PSC (n = 10), PSC + UC (n = 10), and UC (n = 10). The expression of all genes are normalized to an endogenous reference 18S rRNA and presented as a relative fold change to controls according to the comparative Ct method (2^−ΔΔCt^). Dots illustrate each patient and lines the mean plus interquartile range (IQR).Statistics: one-way ANOVA followed by Fisher’s PLSD test within the groups; *indicates p-value < 0.05, ** p-value p < 0.01, *** p-value < 0.001.

As miR-506 is also reported to modulate the expression of AE2 and InsP3R3^19^, we evaluated mRNA levels of those genes in the colonic tissue samples. In ascending colons of PSC + UC patients, we observed downregulation of gene expression for both AE2 (46.5% reduction, *p* = 0.03 vs. controls, and 62.2% reduction, *p* = 0.0001 vs. UC, Fig. 2a) and InsP3R3 (50.2% reduction, *p* = 0.05 vs. controls, and 64.1% reduction, *p* = 0.003 vs. UC, Fig. 2b), whereas AE2 and InsP3R3 expression remained unchanged in PSC patients as compared to controls. By contrast, in patients with UC alone, the expression of AE2 was substantially enhanced (*p* = 0.01 vs. controls, and *p* = 0.003 vs. PSC, Fig. 2a). Moreover, in sigmoid colons of all patient groups, AE2 and InsP3R3 mRNA levels were comparable to controls (Fig. 2c,d).

**Figure 3:**
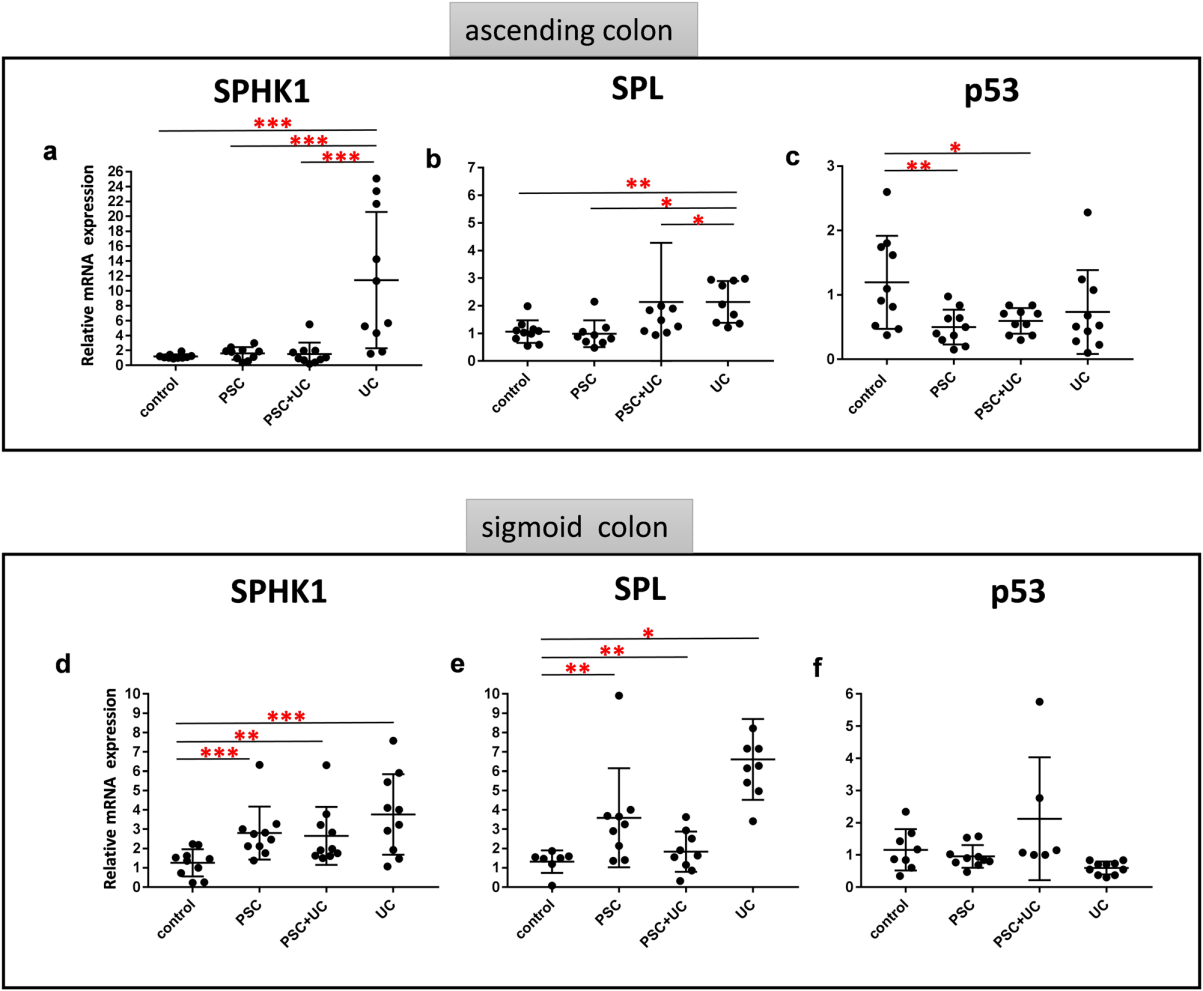
Expression of genes involved in metabolism of sphingosine and p53 in large intestines of the patients. Relative expression levels of sphingosine-kinase-1 (SPHK1) **(a,d)**, sphingosine-1-phosphate lyase (SPL) **(b,e)** and p53 **(c,f)** mRNA in ascending **(a–c)** and sigmoid colons **(d–f)** of controls (n = 10), PSC (n = 10), PSC + UC (n = 10), and UC (n = 10). The expression of all genes are normalized to an endogenous reference 18S rRNA and presented as a relative fold change to controls according to the comparative Ct method (2^−ΔΔCt^). Dots illustrate each patient and lines the mean plus interquartile range (IQR). Statistics: one-way ANOVA followed by Fisher’s PLSD test within the groups; *indicates p-value < 0.05, ** p-value p < 0.01, *** p-value < 0.001 .

On further investigation, we analyzed the expression of genes involved in the metabolism of sphingosine, namely SPHK1 (a predicted miR-506 target) and SPL (a S1P lyase that irreversibly degrades S1P). In those parts of the large intestine where expression of miR-506 was strongly inhibited, the expression of both SPHK1 and SPL were considerably enhanced in comparison to controls. Thus, SPHK1 was extensively upregulated in UC ascending colons (10.4-fold, *p* = 0.009 vs. controls , Fig. 3a), in PSC sigmoid colons (2.5-fold, *p* = 0.004 vs. controls), in PSC + UC sigmoid colons (2.3-fold, *p* = 0.01 vs. controls), and in UC sigmoid colons (3.4-fold, *p* = 0.001 vs. controls; Fig. 3d). Similarly, induction of SPL expression was observed in UC ascending colons (1.9-fold, *p* = 0.002 vs. controls, Fig. 3b), in PSC sigmoid colons (3-fold, *p* = 0.01 vs. controls), in PSC + UC sigmoid colons (1.9-fold, *p* = 0.02 vs. controls), and in UC sigmoid colons (6-fold, *p* = 0.0001 vs. controls; Fig. 3e). In contrast, ascending colons of PSC and PSC + UC patients exhibited SPHK1 and SPL mRNA expression at control levels (Fig. 3a,b). Because SPL is reported to promote apoptosis in response to stress via a p53-dependent pathway and malfunctioning apoptotic pathway is a widespread phenomenon in carcinogenesis^13^ we additionally examined the expression of tumor suppressor protein p53. In ascending colons of PSC and PSC + UC patients, levels of p53 mRNA were substantially suppressed compared to controls (58% reduction, *p* = 0.009 , and 51% reduction, *p* = 0.01, respectively, Fig. 3c), while p53 expression in the other studied groups was comparable to control levels (Fig. 3c,f**)**.

**Figure 4:**
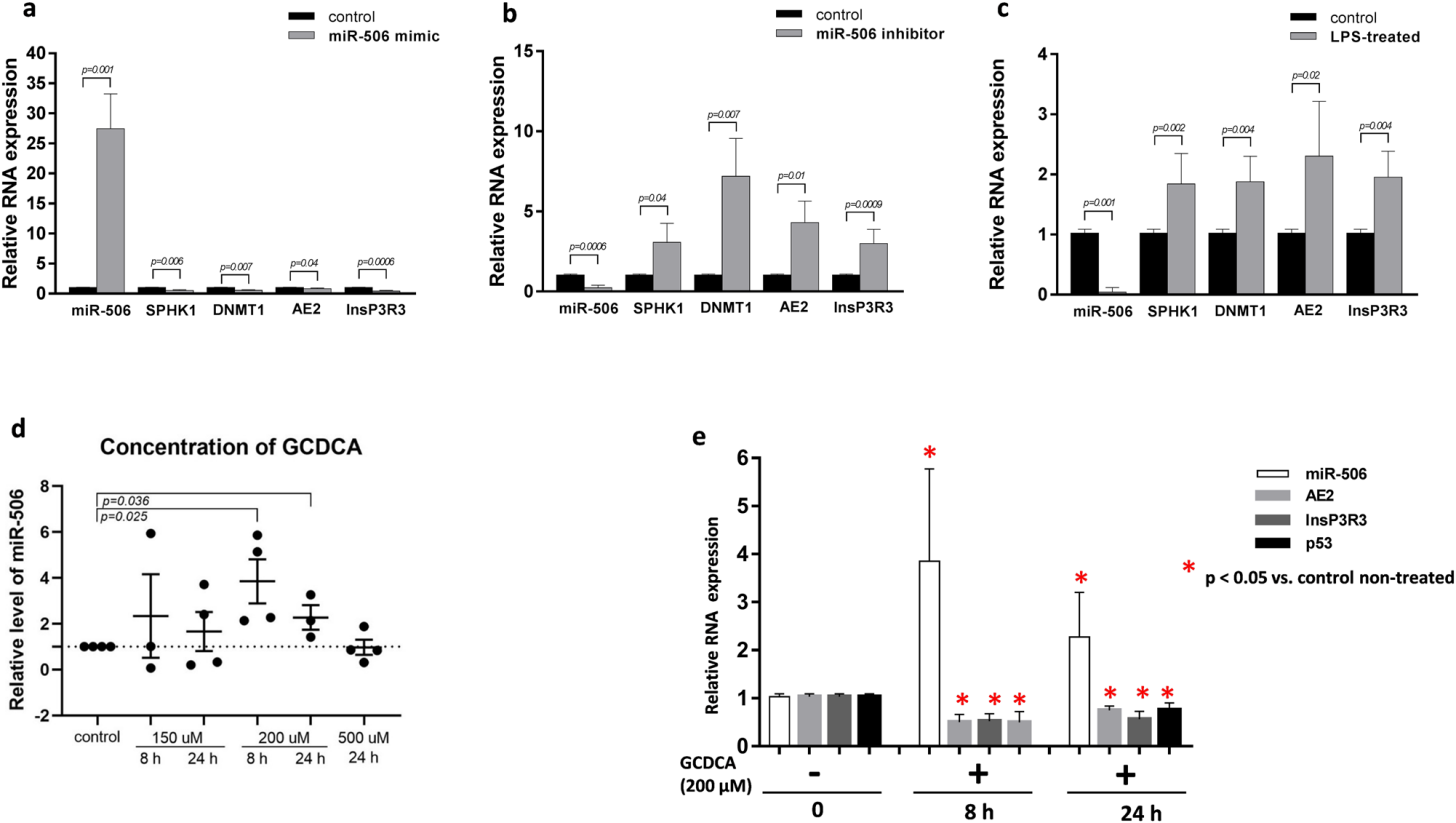
Effects of miR-506 induction and inhibition in Caco-2 cell line. **(a)** Relative expression levels of sphingosine-kinase-1(SPHK1), DNA-methyltransferase-1(DNMT1), anion exchange protein 2 (AE2), and inositol-1,4,5-trisphosphate-receptor (InsP3R3) mRNA after induction of miR-506 by 48-h transfection with mirVana miR506 Mimic (grey bars: cells transfected with miR-506 Mimic; black bars: control non-transfected cells). **(b)** Inhibition of miR-506 by 48-h transfection of Caco-2 cells with mirVana miR506 Inhibitor led to significant upregulation of SPHK1, DNMT1, AE2 and InsP3R3 mRNA levels. (grey bars: cells transfected with miR-506 inhibitor; black bars: control non-transfected cells). **(c)** Lipopolysaccharide (LPS)-induced inflammatory response reduced miR-506 expression and enhanced SPHK1, DNMT1, AE2 and InsP3R3 expressions. **(d)** The effect of glycochenodeoxycholic acid (GCDCA) on miR-506 expression in Caco-2 cells. Dots illustrate each experiment (n = 3–4) and lines the mean plus interquartile range (IQR).The dotted line represents control values. **(e)** GCDCA-induced miR-506 expression suppressed levels of AE2, InsP3R3 and p53 mRNAs both after 8-and 24-h exposure to GCDCA (200 µM). Each experiment was repeated three or four times with similar results. Relative data normalized to endogenous reference genes (miRNA-191 or 18S rRNA) are presented as the mean ± standard error (SE). Statistics: one-way ANOVA followed by Fisher’s PLSD test within the groups *indicates p-value < 0.05 vs. non-treated controls.

Furthermore, we examined the relationship between miR-506 and its target genes in human intestinal epithelial cell lines (Caco-2). Transfection of a modified double-stranded RNA that mimics endogenous miR-506 effectively induced miR-506 activity (27-fold increase, *p* = 0.001 vs. controls) and simultaneously suppressed SPHK1 (35% reduction, *p* = 0.006 vs. controls), DNMT1 (34% reduction, *p* = 0.007 vs. controls), AE2 (22% reduction, *p* = 0.04 vs. controls), and InsP3R3 (59% reduction, *p* = 0.0006 vs. controls), as shown in Fig. 4a.

MirVana miR-506 inhibitor effectively suppressed miR-506 in Caco-2 cells (88% reduction *p* = 0.0006 vs. controls) and concomitantly enhanced the levels of SPHK1 (3-fold, *p* = 0.04 vs. controls), DNMT1 (7-fold, *p* = 0.007 vs. controls), AE2 (4.3-fold, *p* = 0.01 vs. controls), and InsP3R3 mRNA (3.4-fold, *p* = 0.0009 vs. controls, Fig. 4b). Moreover, a pro-inflammatory response was induced in Caco-2 cells by treatment with lipopolysaccharide (LPS). The experiments showed that LPS-induced inhibition of miR-506 (*p* = 0.001 vs. controls) led to upregulation of SPHK1 (1.8-fold, *p* = 0.004), DNMT1 (1.9-fold, *p* = 0.004), AE2 (2.3-fold, *p* = 0.02), and InsP3R3 (1.9-fold, *p* = 0.004, all *p* vs. control non-treated cells Fig. 4c). In addition, we investigated whether conjugated bile acids (for which levels are increased in feces of patients with cholestatic liver diseases^23^) modulate expression of miR-506 in Caco-2 cells. Glycochenodeoxycholic acid (GCDCA, 200 µM) markedly increased miR-506 levels after 8-h and 24-h exposure (2.3-fold vs. controls, *p* = 0.03, and 3.8-fold vs. controls, *p* = 0.02, respectively; Fig. 4d). GCDCA-induced upregulation of miR-506 led to suppression of AE2, InsP3R3, and p53 both after 8 h (52% reduction, *p* = 0.01, 51% reduction, *p* = 0.007, and 52% reduction *p* = 0.01, respectively), and after 24 h (24% reduction *p* = 0.02, 48% reduction, *p* = 0.007, and 26.4% reduction, *p* = 0.03, respectively; all *p* vs. control non-treated cells, Fig. 4e). Additionally, we tested whether drugs routinely used in the treatment of PSC and UC can modify colonic levels of miR-506. Importantly, neither ursodeoxycholic acid (UDCA) nor 5-aminosalicylic acid (5-ASA), nor both used simultaneously, modulated miR-506 expression in Caco-2 cells (data not shown).

**Figure 5:**
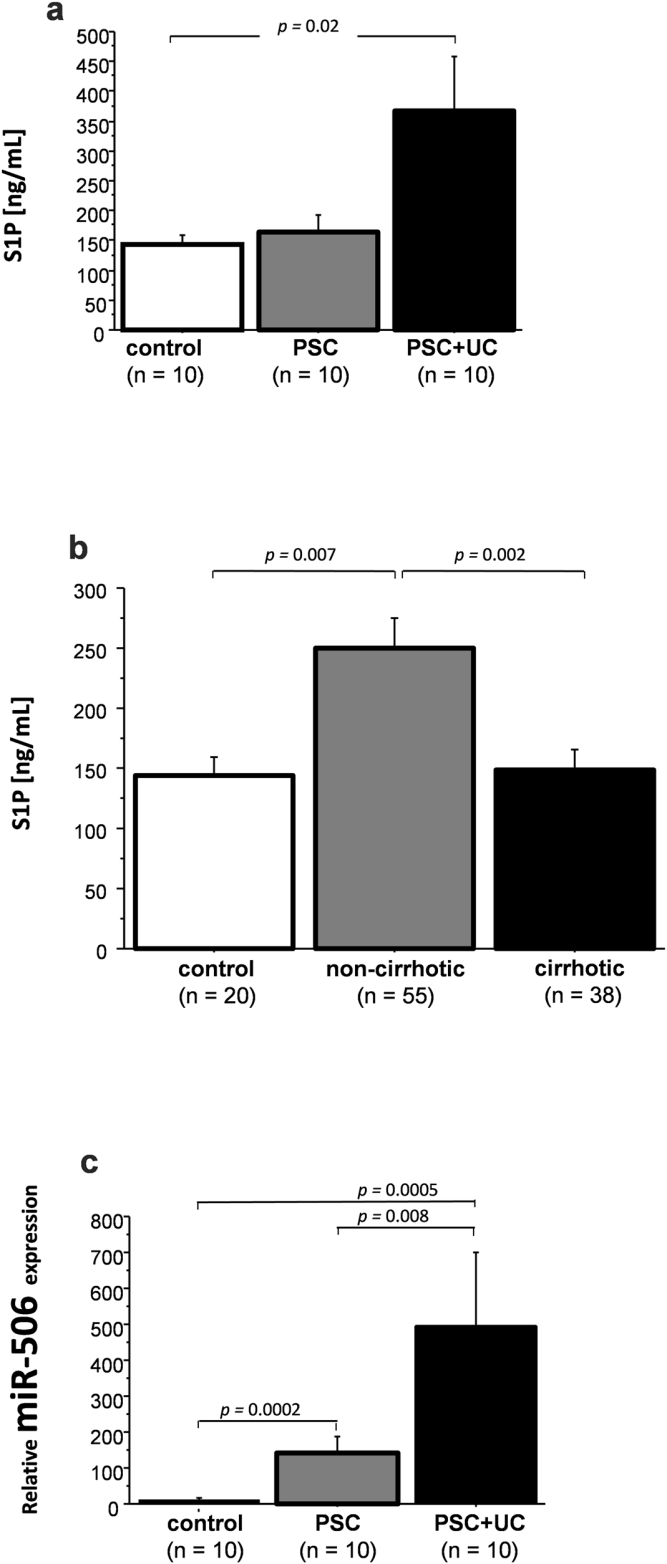
Concentrations of active S1P and relative expression of miR-506 in serum.** (a)** Circulating concentration of sphingosine-1-phosphate (S1P) in serum of PSC and PSC + UC patients in whom colonic tissue were analyzed. **(b)** Serum concentration of S1P in controls, non-cirrhotic PSC and in PSC patients with liver cirrhosis. **(c)** The relative expression of miR-506 in sera of PSC and PSC + UC patients. Data were compared with the Fisher’s (PLSD) test and p-values below 0.05 were considered statistically significant. Bars indicate the mean ± standard error (SE).

Finally, we measured serum levels of S1P. The results showed that in contrast to patients with PSC in these with PSC + UC the concentration of S1P was significantly increased (316 ± 79 ng/mL in PSC + UC vs. 182 ± 50 ng/mL in healthy controls, *p* = 0.02, Fig. 5a). In addition, serum S1P levels showed a significant positive correlation with transaminases: ALT (Rho = 0.7, *p* = 0.04) and AST (Rho = 0.9, *p* = 0.003), but not with markers of cholestasis such as ALP or GGT. We extended our analysis with additional sera from a larger cohorts of 93 patients including 52 patients with PSC and 41 patients with PSC + UC. This analysis confirmed our initial observation and showed the correlation between serum ALT and S1P concentration (Rho = 0.3, *p* = 0.04) in PSC + UC patients. We also noticed that in this group of patients (n = 93) the circulating levels of S1P was higher in non-cirrhotic in comparison to cirrhotic patients with PSC (250 ± 25 ng/mL in non-cirrhotic PSC vs. 149 ± 17 ng/mL in cirrhotic PSC, *p* = 0.0016, or vs. 143 ± 16 ng/mL in healthy controls, *p* = 0.007, Fig. 5b). In the group of patients whose colon tissues were examined, serum expression of miR-506 was substantially induced in both PSC and PSC + UC patients (250-fold increase, *p* = 0.0002 vs. controls and 500-fold increase *p* = 0.008 vs. controls (Fig. 5c). The laboratory markers of the disease severity failed to have any association to the serum miR-506.

## Discussion

In this study, we demonstrated that the level of miR-506 in the ascending colon is a factor that clearly differentiates patients with PSC + UC from patients with UC alone. Furthermore, this study showed that distinctive expressions of miR-506, targeting genes such as SPHK1, DNMT1, SPL, p53, AE2, or InsP3R3, may be responsible for an increased risk of bowel cancer or severe inflammation. Additionally, the functional studies in Caco-2 cells validated the observations from colonic tissues in showing that treatment with GDCA induces, whereas pro-inflammatory activation triggered by LPS, suppresses miR-506 expression.

The expression of miR-506 was substantially increased in the ascending colons of PSC and PSC + UC patients in contrast to UC patients, in whom miR-506 was significantly suppressed (90% reduction) in both the ascending and sigmoid colons. Interestingly, the increased levels of miR-506 were seen in the parts of the colon with the highest concentrations of secondary bile acids (including lithocholic and deoxycholic acids)^24^; reportedly, a majority of PSC patients with UC who develop CRC have tumors located in the right proximal ascending colon, unlike patients with UC alone, whose tumors more frequently occur in the left side of the colon^25^.

MiR-506 has been reported to suppress DNMT1 expression at both the RNA and protein levels in CRC^8^, and it is now widely accepted that DNA methylation plays a key role in silencing numerous cancer-related genes^26^. Interestingly, significant differences in DNMT1 expression were apparent between the groups in our study, and DNMT1 expression negatively correlated with miR-506 expression. Thus, in the ascending colons of UC patients and in the sigmoid colons of all patients, DNMT1 mRNA levels were upregulated, whereas DNMT1 was suppressed in the ascending colons of PSC + UC patients, as compared to controls. The negative correlation between miR-506 and DNMT1 mRNA was additionally confirmed in Caco-2 cell lines. These results are in agreement with previous studies in which the expression of DNMT1 was significantly increased in UC-related carcinogenesis, compared to non-inflammatory colorectal carcinogenesis^27^. DNMT1 is known to be involved in the hypermethylation of the PTEN promoter, what leads to the inhibition of this tumor suppressor gene^15^. A loss of PTEN function is strongly associated with adverse oncological outcomes; furthermore, PTEN affects tumor growth through modulation of the immune response and tumor microenvironment^17^.

Our study clearly demonstrated that in contrast to UC patients, overexpression of miR-506 in the ascending colon of PSC + UC patients (which was accompanied by decreased levels of DNMT1) resulted in upregulation of the PTEN gene and induction of the cyclin-dependent kinase inhibitor p27(Kip1). Hence, the mechanism protecting from neoplasia was activated as a consequence of enhanced expression of suppressor genes, including PTEN and p27(Kip1). These results are in agreement with previous studies, in which induction of PTEN activity led to induction of the cyclin-dependent kinase inhibitor p27(Kip1) in the HT29 cell line^18^. These unexpected results from our study, given that ascending colons of PSC + UC patients are predisposed to neoplasia, suggest that other factors are responsible for the higher rates of CRC in ascending colons with PSC + UC.

An increased delivery of bile acids (BA) or a shift to a more hydrophobic profile of colonic BA could be of importance in the development of CRC. Strong correlations between CRC incidence and levels of fecal bile acids have been observed, and tumor formation is stimulated by enhanced levels of bile acids in in vivo models^28^. Pathological conditions, particularly cholestasis, can lead to exposure of colonic epithelial cells to higher concentrations of secondary hydrophobic bile acid. Interestingly, in the ascending colons of PSC patients where epithelial cells are exposed to elevated levels of secondary BA, the expression of miR-506 was significantly enhanced. The secretion of HCO3− (via inositol trisphosphate-mediated Ca2^+^ release) and the induction of Cl−/ HCO3− exchange (via AE2) serves as a protective mechanism against the presence of toxic bile acids in the colon^20,21^. Our study showed that in the ascending colons of PSC patients, expression of both AE2 and InsP3R3 was significantly suppressed. This suggests that a shield against the presence of toxic bile acids may have been disrupted. In H69 human cholangiocytes, overexpression of miR-506 leads to inhibition of AE2 and InsP3R3, which results in reduction of Cl^−^/ HCO3^−^ exchange activity^29^. Conversely, inhibition of miR-506 by anti-miR-506 enhances AE2 activity and improves Cl^−^/HCO3^−^ exchange^19^. Our in vitro functional studies in Caco-2 cells demonstrated that GCDCA-induced expression of miR-506 resulted in a subsequent inhibition of InsP3R3 and AE2 genes . InsP3R3 degradation has been reported in prostate cancer cells, and decreased apoptosis has been observed under conditions that enhance InsP3R3 degradation^30^. Correspondingly, experimentally induced inhibition of miR-506 by LPS treatment or by transfection with miR506 inhibitor led to upregulation of InsP3R3 and AE2 genes in those cells.

We suggest that the different phenotypic presentation of colitis in PSC + UC versus UC alone may be related to miR-506 expression. In colonic tissue of patients with UC, miR-506 inhibition results in enhanced production of sphingosine-1-phosphate, which leads to production of the active biolipid, sphingosine. Thus, the upregulation of SPHK1 in sigmoid colons of all patients may result in the accumulation of S1P, which is known to be responsible for inflammatory processes^9^. The functional in vitro analysis in Caco-2 cell lines showed that LPS-induced inflammation led to reduced miR-506, which was accompanied by upregulation of SPHK1 mRNA. Our findings are consistent with results from studies in CRC tissues showing that lower levels of miR-506 are associated with increased expression of SPHK1^5^, and activation of SPHKs increase intracellular S1P^10^. Of note, we observed a higher serum concentration of S1P in patients with PSC with concurrent active ulcerative colitis. In those patients serum S1P concentration correlated with ALT, which is a well-established and commonly used marker of hepatic inflammation. Moreover, the increased circulating levels of S1P were observed in non-cirrhotic patients in whom intrahepatic and extrahepatic bile ducts injuries are associated with enhanced inflammation compared to cirrhotic patients who, in contrast, usually have burned down advanced fibrosis with not much inflammatory activity. Whether serum S1P concentration has a potential to be used as a diagnostic marker of PSC + UC needs further investigation in a larger group of patients.

Moreover, upregulation of the SPL enzyme in both parts of the colons of UC patients and in the sigmoid colons of PSC patients (with and without UC), but not in ascending colons of PSC patients, implies a distinctive pathogenesis of inflammation and/or carcinogenesis. It is noteworthy that SPL, which irreversibly degrades the bioactive S1P, is abundantly expressed in enterocytes but downregulated in colon cancer^31^. SPL prevents colon cancer, as cellular accumulation of S1P results in cell transformation^9,32^. Intestinal SPL has been shown to play a protective role in colitis-associated cancer, and reduced SPL levels have been observed in human colon cancer tissues^13^. Interestingly, SPL has been identified as a dual modulator, as it not only influences the metabolism of S1P but also induces cell death under stress conditions^33^. SPL expression is responsive to DNA damage and induces apoptosis through a p53-dependent mechanism^13^. In our study, a lack of induction of SPL expression in ascending colons of PSC + UC patients was associated with a substantial suppression of p53. Moreover, we demonstrated that an exposure of Caco-2 cells to higher concentration of bile acid (GCDC 200 µM) led to the inhibition of p53 gene. Thus, ascending colons of PSC + UC patients, where elevated levels of secondary BA are observed the p53 signaling pathway—which is considered fundamental for tumor suppression—may not be adequately activated.

This study has some limitations related to unavailability of biological material. These include lack of experiments performed on colorectal cancer tissue, lack of analyses on sera from patients with ulcerative colitis without PSC and lack of experiment on protein levels of miR-506 in the colonic tissue. However despite of these shortcomings we believe obtained data has a potential to stimulate further studies in this so clinically important area.

**Figure 6:**
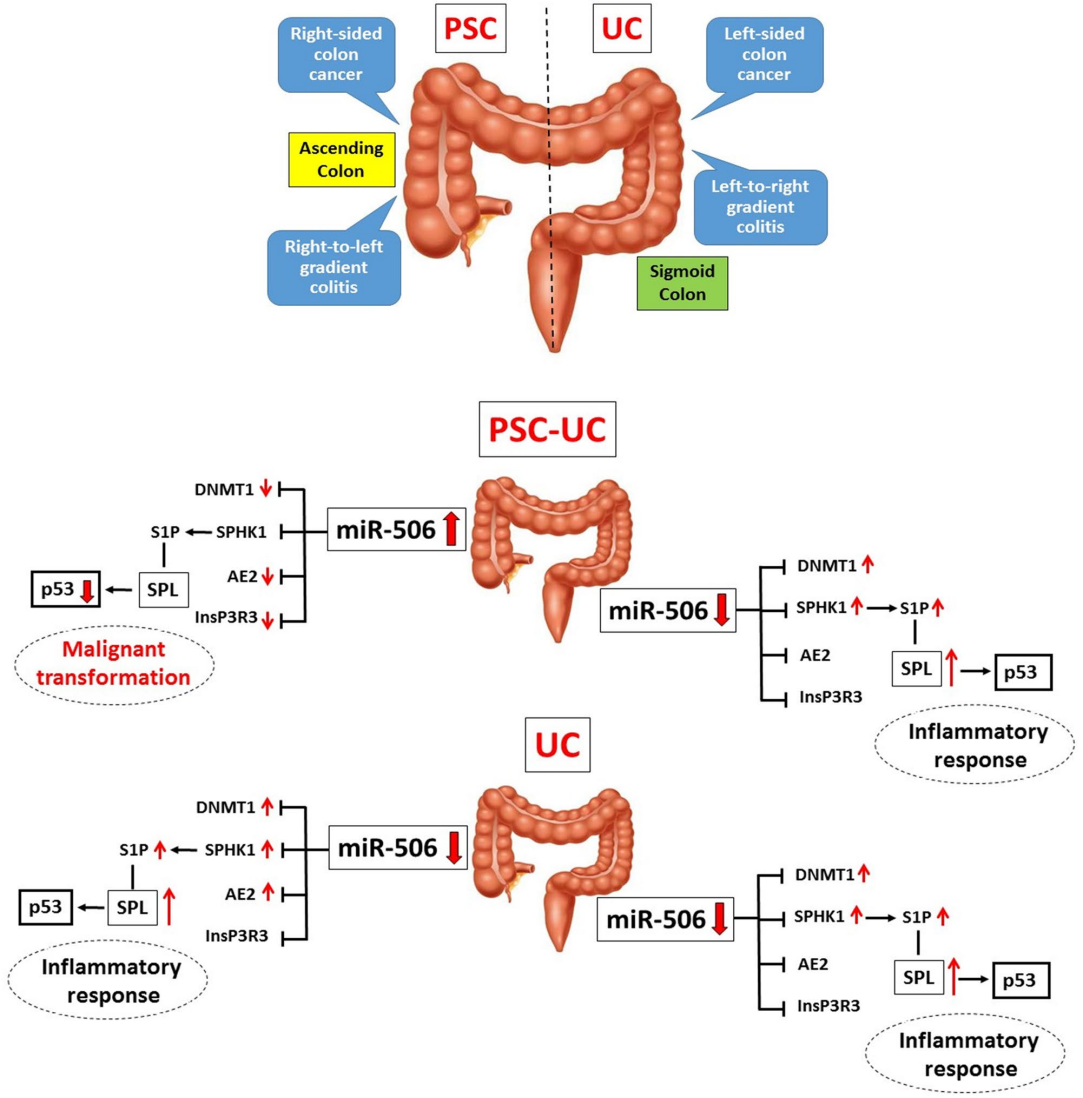
Schematic figure showing the possible contribution of miR-506 to phenotypic presentation of colitis in patients with PSC + UC compared to those with UC alone. MicroRNA-506 expression was upregulated in ascending colons of patients with primary sclerosing cholangitis and concurrent ulcerative colitis (PSC + UC). This overexpression of miR-506 resulted in reduced expression of anion exchange protein 2 (AE2) and inositol-1,4,5-trisphosphate-receptor (InsP3R3), which destabilized protection against toxic bile acids. The lack of sphingosine-kinase-1 (SPHK1) induction in the face of increased inflammation in this part of the colon, as well as the loss of sphingosine-1-phosphate lyase (SPL) activity, promoted neoplastic transformation via a p53-dependent pathway. In contrast, colonic suppression of miR-506 in both parts of UC patient colons, and in the sigmoid colons of PSC + UC patients, was accompanied by a substantial increase in SPHK1 expression. The enhanced levels of kinase SPHK1 resulted in upregulation of bioactive sphingosine-1-phosphate (S1P) which led to further activation of S1P-dependent signaling pathways. The net effect of these responses is severe inflammation. S1P is continuously and irreversibly degraded by the SPL enzyme, which is abundantly expressed in enterocytes, protecting from colitis-associated cancer.

In summary, our results suggest that a different phenotypic presentation of colitis in PSC may be related to epigenetic modulations via miR-506 (Fig. 6). MiR-506 downregulation led to enhanced production of S1P (via SPHK1 induction), which initiates colonic inflammatory responses. The absence of enhanced SPL expression and the inhibition of the p53 tumor suppressor gene might explain the differences in pathogenesis of CRC in PSC + UC versus UC patients. We believe the role of miR-506 expression deserves further investigation as modulation of miR-506 may be a potential target to hamper inflammation or possible cancer development in colonic mucosa.

## Methods

### Patient characteristics

The study included four groups of patients: PSC patients (n = 10) who had never been diagnosed with concomitant IBD; PSC + UC patients (n = 10) showing the macroscopic features of UC in colonoscopy, which were confirmed with histology examination; UC-only patients (n = 10); and healthy controls who underwent colonoscopies for various indications and showed neither macroscopic nor microscopic abnormalities in their colons (n = 10). Biopsies from the ascending and sigmoid colons, as well as blood samples, were collected from all patients during surveillance colonoscopies. From each patient, a corresponding colon mucosa sample (2–3 mm) was taken from pre-defined sites in the intestine. Immediately after isolation, the biopsies were placed in RNAlater Solution (Ambion/Applied Biosystems, Foster City, CA). All samples were kept at − 80 °C until RNA extraction. Demographic and laboratory features of the participants are presented in Table 2. As we have noticed a difference in serum concentrations of S1P between PSC and PSC + UC patients we have extended our analysis with additional sera from PSC (n = 52), and PSC + UC patients (n = 41), and gender- and age-matched controls (n = 20). Demographic and laboratory features of those subjects are presented in Table 3. All patients with PSC were treated with UDCA (15 mg/kg), whereas PSC + UC patients additionally received 5-ASA (2–3 g/daily).

**Table 2:** Demographic and laboratory features of patients in whom colonic tissue, S1P serum concentration and miR-506 serum expression were analyzed.

	Control (n = 10)	PSC (n = 10)	PSC + UC (n = 10)	UC (n = 10)
Gender (male/female)	6/4	8/2	8/2	2/8
Age (years)	50 ± 4	32 ± 14	32 ± 14	43 ± 17
Hb (mg/dl, normal F: 12–16, M: 14–18)	ND	14 ± 0.5	13 ± 1.7	ND
Bilirubin (mg/dl, normal < 1.1)	ND	0.7 ± 0.2	2.0 ± 0.7	0.4 ± 0.2
ALP (IU/l, normal 30–120)	ND	214 ± 39	381 ± 105	80 ± 24
GGTP (IU/l, normal F < 66, M < 100)	ND	366 ± 118	484 ± 232	17 ± 13.3
ALT (IU/l, normal < 40)	ND	152 ± 36	81 ± 23	15 ± 8.4
Cirrhosis (yes/no)	N/A	2/8	3/7	N/A

Values are given as mean ± SD, unless stated otherwise.

*PSC* primary sclerosing cholangitis, *UC* ulcerative colitis, *Hb* hemoglobin, *ALP* alkaline phosphatase, *GGTP* gamma-glutamyl transferase, *ALT* alanine aminotransferase, *SD* standard deviation, *N/A* not applicable, *ND* no data.

**Table 3:** Demographic and laboratory features of patients in whom only S1P serum concentration were analyzed.

	Control (n = 20)	PSC (n = 52)	PSC + UC (n = 41)
*Gender (male/female)*	19/1	31/21	32/9
*Age (years)*	58 ± 4	32 ± 2	33 ± 2
Hb (mg/dl, normal F: 12–16, M: 14–18)	ND	12.6 ± 0.3	12.7 ± 0.3
Bilirubin (mg/dl, normal < 1.1)	ND	4.1 ± 0.9	2.5 ± 0.5
ALP (IU/l, normal 30–120)	ND	298 ± 30	318 ± 33
GGTP (IU/l, normal F < 66, M < 100)	ND	228 ± 32	260 ± 49
ALT (IU/l, normal < 40)	ND	87 ± 9	87 ± 12
Cirrhosis (yes/no)	N/A	16/36	22/19

Values are given as mean ± SD, unless stated otherwise.

*PSC* primary sclerosing cholangitis, *UC* ulcerative colitis, *Hb* hemoglobin, *ALP* alkaline phosphatase, *GGTP* gamma-glutamyl transferase, *ALT* alanine aminotransferase, *SD* standard deviation, *N/A* not applicable, *ND* no data.

### RNA and miRNA expression analysis

Total RNA from colon tissues was isolated using RNeasy Mini Kit (Qiagen, Germany) which provided fast purification of high-quality total RNA (the ratios of the absorbance values of 260 nm vs. 280 nm (A260/A280) were 1.94–2.09, with most samples yielding up to 310 ng/µL of RNA. RNA extraction from 200 μL human serum was carried out with miRNeasy Serum/Plasma Advanced Kit (Qiagen). The synthesis of first-strand cDNA was carried out using SuperScript II RT (Invitrogen, USA), according to the protocol previously described^22^. Expressions of specific genes were measured by 7500 Fast Real-Time PCR System (Applied Biosystems, USA) using human TaqMan^®^ Gene Expression Assays for: SPHK1 (Hs00184211_m1), DNMT1 (Hs00945875_m1), SPL1 (Hs00393705_m1), PTEN (Hs02621230_s1), p27^(Kip1)^ (Hs00153277_m1), p53 (Hs01034249_m1), AE2 (Hs01586776_m1), InsP3R3 (Hs01573555_m1), and 18SRNA (Hs99999901_s1). All used TaqMan MGB probes spanned an exon-exon junction for excluding genomic DNA as a template in a real-time PCR reaction. Eukaryotic 18S rRNA endogenous control was quantified as a means of correcting nucleic acid loading differences. Mean cycle threshold (Ct) values for all genes were quantified with the Sequence Detection software (Applied Biosystems).The amount of target, normalized to an endogenous reference and relative to the expression levels in healthy controls were determined using the 2^−ΔΔCt^ formula.

MiR-506 cDNA synthesis was carried out using TaqMan Advanced miRNA cDNA Synthesis Kit (Applied Biosystems, USA) according to the manufacturer’s protocol, and expression of miR-506 (Assay ID 478958_mir) and reference microRNA miR-191 (477952_mir) were measured using TaqMan Advanced miRNA assays (Applied Biosystems, USA).

### ELISA

Serum concentrations of sphingosine-1-phosphate were measured using a human ELISA Kit (CEG031Ge, Cloud Clone Corp, USA) according to the manufacturer’s instructions.

### Cell culture and treatments

Caco-2 lines of heterogeneous human epithelial colorectal adenocarcinoma cells from American Type Culture Collection (American Type Culture Collection, ATCC) were grown according to the manufacturer’s protocol. We used ready-to-transfect molecules that specifically induce miR-506: mirVana miRNA Mimic and Inhibitor (hsa-miR-506-3p; ID:MC10709; ThermoFisher Scientific). Transient transfection of Caco-2 cells was performed using Lipofectamine RNAiMAX (Invitrogen, USA) for 48 h. Caco-2 were cultured for 24 h in the presence or absence of UDCA (50 and 150 µM; U5127-1G, Sigma-Aldrich) and 5-amino-2-hydroxybenzoic acid (500 and 1000 µM; Mesalamine A3537, Sigma-Aldrich), or simultaneously treated with UDCA and 5-ASA. To initiate the inflammatory process, cells were incubated in Eagle's Minimum Essential Medium (EMEM) containing a lipopolysaccharide (5 µg/ml) (LPS, L4391-1MG SIGMA). To investigate influence of GCDCA on miR-506 expression, Caco-2 cells were incubated with either 150, 200, or 500 µM GCDCA (sodium glycochenodeoxycholate, Sigma-Aldrich, ID: 24895023) for 8 or 24 h. All experiments were repeated at least three or four times.

### Statistics

For statistical analysis and graphical presentation the StatView (SAS Institute Inc, USA) and GraphPad Prism 7 (San Diego, CA) programs were used. Continuous variables were summarized as means (interquartile range [IQR, lowest 25% − highest 25%]) whereas data from in vitro studies are reported as mean of at least 3–4 independent experiments with SEM as error bars. Data were compared with the Mann–Whitney U test or the Fisher Protected Least Significant Difference (PLSD) test. Correlation analyses were performed using the nonparametric Spearman’s rank method. Results were considered statistically significant of *p* < 0.05.

### Ethics declarations

Each patient gave informed consent prior to participating in this study. The research protocol was approved by the Ethics Committee of Pomeranian Medical University (no. BN-001/43/06 and BN-001/122/06) and conformed to the ethical guidelines of the 1975 Declaration of Helsinki.
